# Antischistosomal activity of imidazolidine-2,4-dione derivatives

**DOI:** 10.1128/aac.01748-25

**Published:** 2026-01-26

**Authors:** Anna Jaromin, Anna Czopek, Thainá R. Teixeira, Agnieszka Zagórska, Josué de Moraes

**Affiliations:** 1 Department of Lipids and Liposomes, Faculty of Biotechnology, University of Wrocław 49572, Wrocław, Poland; 2 Department of Medicinal Chemistry, Jagiellonian University Medical College 49573, Cracow, Poland; 3 Research Center on Neglected Diseases, Guarulhos University 92928, Guarulhos, State of São Paulo, Brazil; 4 Research Center on Neglected Diseases, Scientific and Technological Institute, Brazil University 149945 , São Paulo, State of São Paulo, Brazil; The Children's Hospital of Philadelphia, Philadelphia, Pennsylvania, USA

**Keywords:** imidazolidine-2,4-dione derivatives, antiparasitic properties, *Schistosoma mansoni*, schistosomiasis

## Abstract

Schistosomiasis is a neglected tropical disease with limited treatment options and growing concerns over praziquantel resistance. We evaluated a series of 12 synthetic compounds for antiparasitic activity against *Schistosoma mansoni* adult worms *in vitro*. Isoindole-1,3-dione derivatives were inactive, whereas four imidazolidine-2,4-dione derivatives displayed selective antischistosomal activity with low cytotoxicity in mammalian cells. These findings highlight imidazolidine-2,4-dione as a promising scaffold for the development of new therapeutic agents against schistosomiasis.

## INTRODUCTION

Schistosomiasis is a neglected tropical disease caused by parasitic flatworms of the genus *Schistosoma*. It affects approximately 250 million people worldwide, with significant morbidity and socioeconomic burden in endemic regions, particularly in sub-Saharan Africa, South America, and parts of Asia (1). Praziquantel, a synthesized pyrazine isoquinoline derivative, is currently the only widely used drug, and the risk of resistance has prompted the search for new chemical entities (2, 3).

To address these challenges and meet the United Nations Sustainable Development Goals, the World Health Organization has established a 2021–2030 roadmap for the control and elimination of neglected tropical diseases (4). A key pillar of this roadmap is the urgent need for the discovery and development of novel therapeutic agents targeting schistosomiasis. In this context, the identification of new chemical scaffolds with selective anthelmintic activity is a critical step toward diversifying the pipeline and reducing reliance on a single therapeutic option (5, 6).

Drug repurposing offers a cost-effective strategy to combat neglected tropical diseases, utilizing existing drugs with known safety profiles to expedite clinical use. Another innovative strategy is hybrid drug discovery, which merges pharmacophores from different bioactive molecules (7, 8). The goal is to enhance antischistosomal efficacy, reduce resistance, and enable multi-stage parasite targeting. By integrating distinct bioactive fragments into a single scaffold, these hybrids seek to overcome praziquantel’s limitations.

**Fig 1:**
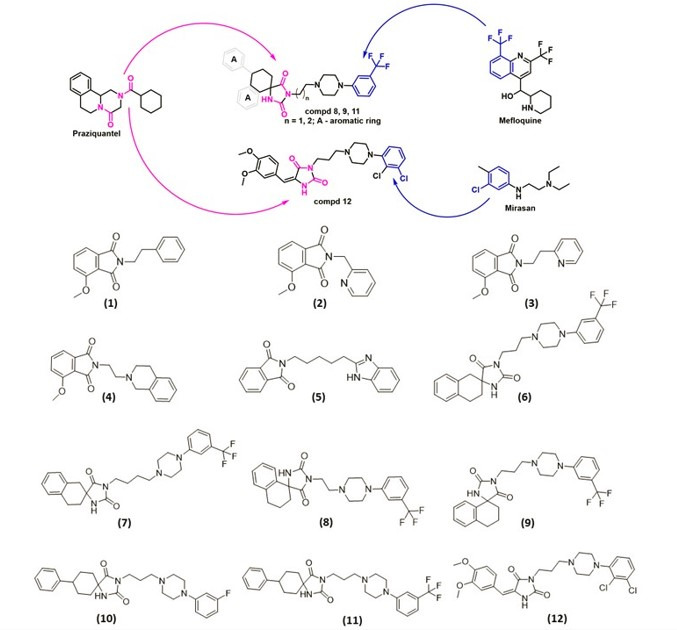
Hybrid scaffold design strategy and chemical structures of the compounds evaluated in this study. (Top) Schematic representation of the pharmacophore-based design combining structural fragments from praziquantel, mefloquine, and mirasan to generate imidazolidine-2,4-dione derivatives (compounds **8**, **9**, **11**, and **12**). (Bottom) Chemical structures of all synthesized compounds (1–12), including isoindole-1,3-dione derivatives (1–5) and imidazolidine-2,4-dione derivatives (6–12). Compound **8** is novel; all other compounds correspond to previously reported structures. Full synthetic details and analytical data are provided in the supplemental material.

Herein, we present a hybrid design strategy that combines pharmacophoric elements from praziquantel, mirasan, and mefloquine to generate novel scaffolds with potential antischistosomal activity (Fig. 1). The rationale for compound selection was based on hybridization of pharmacophoric fragments derived from these molecules, representing distinct chemotypes previously associated with antiparasitic activity (9, 10). Praziquantel provides a rigid hydrophobic core and cyclic amide motif that interacts with calcium channels, whereas mirasan serves as a structural precursor for the development of hycanthone and oxamniquine, two classical DNA-intercalating antischistosomal agents (11, 12). Mefloquine contributes a quinoline-based lipophilic scaffold that perturbs parasite membrane stability (13). The imidazolidine-2,4-dione and isoindole-1,3-dione frameworks were chosen as versatile linkers to integrate these pharmacophores, enabling modulation of electronic and steric properties within a chemically accessible platform. All final compounds (**1–7** and **9–12**), except for compound **8**, have been previously reported (9, 10, 14–17). The detailed description of the synthesis of compounds **1–12** is presented in the supplemental material (available at http://dx.doi.org/10.60731/ujcm.18122501).

Adult *Schistosoma mansoni* worms (BH strain) were obtained from infected Swiss mice 49 days post-infection (18).

Each experiment was performed in triplicate using four pairs of adult worms per well, incubated in 24-well plates with 2 mL of RPMI 1640 medium supplemented with 10% fetal bovine serum at 37°C in a 5% CO_2_ atmosphere. In the initial screening, all compounds were tested at 50 µM for 72 h, and worm viability was assessed at 0, 24, 48, and 72 h under an inverted microscope. Parasite death was defined as the complete absence of movement for at least 1 minute following gentle mechanical stimulation with fine forceps (19). Compounds that reduced worm viability in this primary screen were subsequently tested across concentrations ranging from 6.25 to 50 µM to determine the 50% effective concentration (EC_50_) by non-linear regression of concentration–response data using GraphPad Prism 10 (20, 21). For cytotoxicity assays, Vero cells (1 × 10⁴ cells/well) were incubated with each compound for 24 h prior to MTT addition, and absorbance was measured at 570 nm (22). All experiments were performed in triplicate, and results are expressed as mean ± SD from three independent replicates.

Representative dose–response curves for the active compounds are provided in Fig. S1 (http://dx.doi.org/10.60731/ujcm.18122501). All active molecules induced progressive, concentration-dependent reductions in worm motility and viability without immediate paralysis, suggesting a slower onset of action distinct from the rapid spastic contraction produced by praziquantel. These observations support the hypothesis that the imidazolidine-2,4-dione scaffold acts through a mechanism different from that of praziquantel.

All compounds from the isoindole-1,3-dione series (**1–5**) were inactive at the tested concentration, despite variations in the substituents on the isoindole-1,3-dione ring (e.g., OCH_3_ or H) and differences in the length of the linker connecting the isoindole-1,3-dione to the amine moiety (containing pyridine, benzimidazole, or 1,2,3,4-tetrahydroisoquinoline groups). Among the imidazolidine-2,4-dione derivatives (**6–12**), compounds **6**, **7**, and **10** were also inactive. In contrast, compounds **8**, **9**, **11**, and **12** induced marked reductions in worm viability (Table 1).

**TABLE 1:** *In vitro* antischistosomal activity, cytotoxicity, and selectivity indices of synthetic compounds **1–12^*a*^***^,^*^*c*^

Compound	*S. mansoni* EC_50_ (μM)	Vero cells CC_50_ (μM)	SI
1	>50	ND	ND
2	>50	ND	ND
3	>50	ND	ND
4	>50	ND	ND
5	>50	ND	ND
6	>50	ND	ND
7	>50	ND	ND
8	18.1 (17.2–22.5)^*b*^	>200	>11.05
9	12.6 (9.7–16.4)	>200	>15.87
10	>50	ND	ND
11	19.8 (17.6–23.9)	>200	>10.1
12	13.3 (10.1–17.3)	>200	>15.03
PZQ	0.98 (0.7–1.2)	>200	>204

^*a*^ CC_50_, 50% cytotoxic concentration against Vero cells; EC_50_, 50% effective concentration based on mortality of *S. mansoni* adult worms; ND, not determined; PZQ, praziquantel; SI, selectivity index (CC_50_/EC_50_).

^*b*^ 95% confidence interval.

^*c*^ Values represent means from three independent experiments, each performed in triplicate.

It is worth mentioning that **8**, **9**, and **11** have an imidazolidine-2,4-dione scaffold with a α-tetralinyl or 4-phenylcyclohexyl moiety, connected via an ethyl or propyl linker to a 3-trifluoromethylphenylpiperazine moiety. This structure–activity relationship analysis revealed that replacing the α-tetralin scaffold with β-tetralin (**6** vs **9**) or replacing the 3-trifluoromethyl group with a 3-fluoro group in the phenylpiperazine moiety (**10** vs **11**) in their structural counterparts resulted in the loss of biological activity against *S. mansoni*. Moreover, the substitution of the 2,3-dimethoxybenzylidene fragment into the imidazolidine-2,4-dione ring, along with the replacement of the 3-trifluoromethyl group with a 2,3-dichloro group (**12**), allowed for the retention of antischistosomal activity. Interestingly, the physicochemical properties of pharmacologically active hybrid compounds were similar (lipophilicity and water solubility), and all compounds agreed with the drug-likeness and Lipinski rule (see Table S1 at http://dx.doi.org/10.60731/ujcm.18122501).

These active compounds (**8**, **9**, **11**, and **12**) were subsequently tested in concentration–response experiments. None of the compounds tested showed cytotoxicity in Vero cells at concentrations up to 200 µM. Consequently, selectivity indices for **8, 9**, **11**, and **12** were all above 10, indicating a favorable selectivity profile. Detailed results for all tested compounds, including EC_50_, 50% cytotoxic concentration against Vero cells, and SI values, are presented in Table 1. Although EC_50_ values in the 10–20 µM range are moderate, they are consistent with reported activity thresholds for early-stage antischistosomal hits (23, 24).

Taken together, these findings support the imidazolidine-2,4-dione scaffold as a promising platform for the development of new antischistosomal agents. The inactivity of the isoindole-1,3-dione series underlines the importance of scaffold selection in the early stages of drug discovery. Compounds **9** and **12**, in particular, demonstrated high potency and selectivity, making them strong candidates for further pharmacological and mechanistic studies.

Further optimization of these hybrid derivatives, including an expanded structure–activity relationship analysis of compound **8** and its analogs, is currently underway in our laboratory. These studies aim to enhance potency while preserving the favorable selectivity and physicochemical characteristics demonstrated in the present work.

## Data Availability

The data that support the findings of this study are available from the corresponding author upon reasonable request.
